# Therapeutic effect of miR-30b-5p-loaded lentivirus on experimental autoimmune uveitis via inhibiting Notch signaling activation

**DOI:** 10.1186/s12967-025-06438-x

**Published:** 2025-04-10

**Authors:** Xuewei Yin, Huixia Wei, Lijie Guo, Bin Liu, Yuan Peng, Mengxian Zhou, Yan Qiu, Ruyi Qu, Yane Gao, Qiuxin Wu, Wenjun Jiang, Hongsheng Bi, Dadong Guo

**Affiliations:** 1 Shandong University of Traditional Chinese Medicine, Jinan, 250002 China; 2 Affiliated Eye Hospital of Shandong University of Traditional Chinese Medicine, No. 48#, Yingxiongshan Road, Jinan, 250002 China; 3 Shandong Provincial Key Laboratory of Integrated Traditional Chinese and Western Medicine for Prevention and Therapy of Ocular Diseases, Jinan, 250002 China; 4 Guangzhou Laboratory, Guangzhou Medical University, Guangzhou, 510000 China; 5 Shandong University of Traditional Chinese Medicine Second Affiliated Hospital, Jinan, 250002 China; 6 Shandong Academy of Eye Disease Prevention and Therapy, Medical College of Optometry and Ophthalmology, Shandong University of Traditional Chinese Medicine, No. 48#, Yingxiongshan Road, Jinan, 250002 China

**Keywords:** Experimental autoimmune uveitis, Notch signaling pathway, miR-30b-5p, Th cell, Notch1, Dll4

## Abstract

**Background:**

Uveitis is a common recurrent autoimmune disease that seriously endangers the visual health of patients. MicroRNAs (miRNAs) are closely related to a series of autoimmune diseases.

**Methods:**

The present study aimed to investigate the effect of miR-30b-5p on experimental autoimmune uveitis (EAU) and its role in Notch signal activation as well as T helper (Th) cell differentiation, the relationship between miR-30b-5p levels and Notch signal activation, as well as Th cell differentiation in uveitis was further explored through flow cytometry, Immunohistochemistry immunofluorescence staining, PCR Array, and Ingenuity Pathway Analysis, and other technical methods to determine the fidelity of miR-30b-5p strategies in treating uveitis in vivo and in vitro*.*

**Results:**

We demonstrated that ocular inflammation was significantly alleviated in EAU rats after miR-30b-5p intervention. miR-30b-5p could effectively inhibit Notch signaling activation and Th17 cell differentiation both in vitro and in vivo, and the Th17/Treg ratios were also notably decreased. Moreover, both Notch signaling and Th17 activation pathways were enriched and activated, in which Notch1 was the upstream regulatory molecule of Dll4 and IL-10 was an up-regulated upstream regulatory network molecule. Furthermore, miR-30b-5p could significantly reduce apoptosis in vitro, and clinical in vitro cell studies have shown that inactivating Notch pathway can improve the imbalance of Th17/Treg and cell apoptosis in T lymphocytes of patients with uveitis.

**Conclusions:**

Together these studies identify that miR-30b-5p can significantly inhibit Notch signaling activation and Th17 cell differentiation, thereby restoring the Th17/Treg balance to treat uveitis, which may provide new insights into treating uveitis using miRNA interfering strategies.

**Graphical Abstract:**

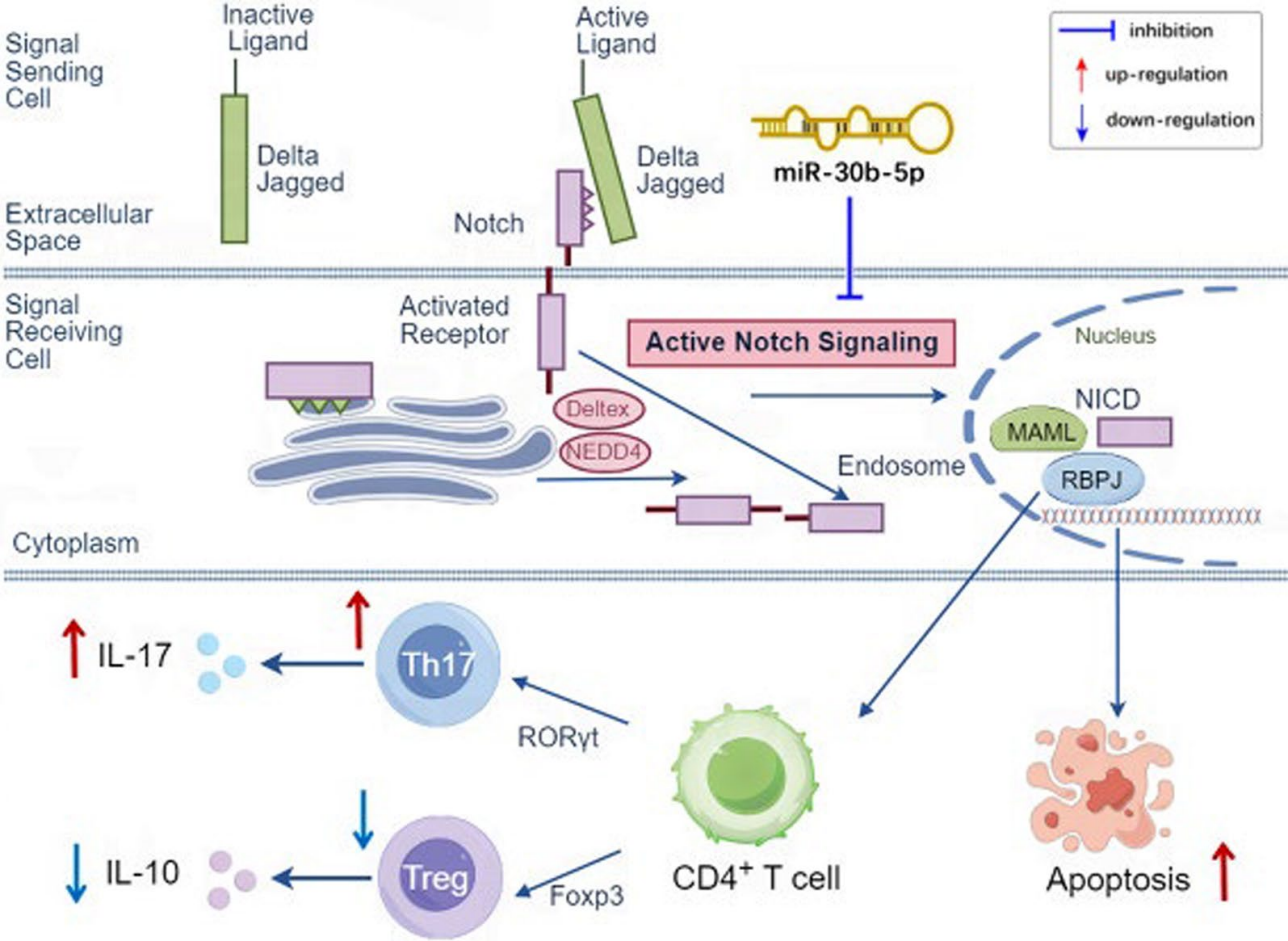

**Supplementary Information:**

The online version contains supplementary material available at 10.1186/s12967-025-06438-x.

## Introduction

Uveitis is a common autoimmune disease that can seriously endanger the visual health of patients. Clinically, there is no exact and reliable treatment for uveitis except hormones or immunosuppressive agents. Therefore, it is of great significance to explore the pathogenesis of uveitis to find new therapeutic targets. The Notch signaling pathway is an essential pathway in regulating autoimmune diseases and inflammatory response, which can play a role in regulating naïve CD4^+^ T cell differentiation and affecting the proportion balance of T helper (Th) cells [1]. RBPJ, a transcription factor, plays an essential role in the Notch signaling activation, and RBPJ inhibitor-1 (RIN1) is an RBPJ inhibitor that can block the functional interaction between RBPJ and SHARP to inactivate the Notch signaling.

MicroRNAs (miRNAs) can negatively regulate the expression of target genes at the post-transcriptional level by RNA interference (RNAi), which is closely related to the course of a variety of autoimmune diseases. It was found that miR-30b can negatively regulate the expression of Notch1 to significantly promote the levels of IL-10 and NO in dendritic cells (DCs) and thus affects the immune response [2]. Tang et al. [3] found that miR-301a-3p is a critical regulator in Th17 cell differentiation, playing an important role in the pathogenesis of rheumatoid arthritis patients; Cruz et al. [4] confirmed that the overexpression of miR-27 in mouse T cells can inhibit the differentiation of regulatory T (Treg) cells, resulting in Treg dysfunction and immune tolerance dysfunction in mice.

The abnormal expression of miRNAs is closely associated with the pathogenesis of uveitis. A Study has revealed that in the peripheral blood of patients with acute anterior uveitis, the levels of miR-146a, miR-143, miR-205, and miR-9-3 significantly reduced, whereas the levels of miR-301a and miR-23a increased significantly, confirming that miRNAs play an important role in the pathogenesis of uveitis[5]; meanwhile, miR-155 can regulate the immune response of Th17 cells by targeting the Ets-1 gene, inhibit the number of pathogenic Th17 cells, thereby reducing the incidence of uveitis patients[6]. Moreover, animal experiments have also shown that miR-146a-5p, miR-155-5p, miR-223-3p, and miR-147b related to the NF-κB signaling pathway are all highly expressed in the iris and ciliary body of uveitis rats, whereas miR-182-5p, miR-183-5p, and miR-9-3p showed decreased levels [7].

miR-30b can down-regulate the expression of Delta-like 4 (Dll4) ligand in the Notch signaling pathway, thereby regulating the development and formation of blood vessels [8]. Our previous study found that the miR-30b-5p levels showed significantly low expression in the spleen, lymph nodes, and eye tissues in EAU rats, suggesting that the downregulated miR-30b-5p is closely related to the pathogenesis of uveitis [9, 10]. In addition, our findings also highlighted that activation of the Notch signaling pathway can exaggerate Th17 and Treg cell differentiation, disrupt the CD4^+^/CD8^+^ and Th17/Treg balance, aggravating the severity of EAU; inactivation of the Notch signaling pathway contributes to the CD4^+^/CD8^+^ and Th17/Treg balance in EAU rats, suggesting that Notch signaling may be a potentially important therapeutic target in clinical practice [11]. In this study, we aimed to investigate the therapeutic effect of miR-30b-5p on EAU and to explore the underlying mechanism of miR-30b-5p regulating the Notch signaling pathway in the pathogenesis of uveitis via Notch signaling-mediated Th cell differentiation. Our findings will provide new insights for elucidating the pathogenesis of uveitis and establishing a new therapeutic approach with miRNA as the target.

## Materials and methods

### Materials

Lymphocyte separating solution (Solarbio, Beijing, China); beta-mercaptoethanol, glutamine (Ameresco, USA); fetal bovine serum (NEST Biotech., Wuxi, China); RPMI 1640 medium (HyClone, USA); fetal bovine serum (NEST, Wuxi, China); FITC-CD4, PE-CD8, PE-IL-17, APC-CD25, PE-Foxp3 (eBioscience, USA); Annexin V-FITC/PI apoptosis detection kit, Fixation/Permeabilization Kit with BD Golgistop™ (BD Biosciences, USA); interphotoreceptor retinoid-binding protein (IRBP) peptide (residues 1177–1191, sequence: ADGSSWEGVGVVPDV), phosphate buffered saline (PBS, pH 7.2) (Sangon Biotech (Shanghai) Co., Ltd., Shanghai, China); Mycobacterium tuberculin H37RA (TB) (Difco, USA); complete Freund's adjuvant (CFA, Sigma, USA); RNA Tissue/Cell Rapid Extraction Kit, BCA protein assay kit and SPARKScript II miRNA 1st strand cDNA synthesis kit (Shandong Sparkjade Biological Co. Ltd., Jinan, China); ChamQ Universal SYBR qPCR Master Mix (Vazyme Biotech Co., Ltd., Nanjing, China); RNeasy Microarray Tissue Mini Kit, DNA Reverse Transcription Kit, 2 × SYBR Green I Kit (QIAGEN, Germany); miR-30b-5p-carrying lentivirus and miR-30b-5p control vector carryng lentivirus (Genomeditech, Shanghai, China); Rabbit monoclonal Notch1 antibody (Abcam, Shanghai, China); rat GAPDH Rabbit pAb, Dll4 antibody Rabbit pAb (Bioss, Beijing, China); human β-actin Rabbit pAb, Dll4 Rabbit pAb, RBPJ Recombinant Rabbit mAb antibody (Bioss, Beijing, China); rat Notch1, Dll4 ELISA Kits (Wuhan ColorfulGene Biological Technology Co., Ltd., Wuhan, China); rat IL-10, IL-17 ELISA kits (Shanghai Jianglai Biological Co. Ltd., Shanghai, China); FITC rat IgG2b κ Isotype Control, PE rat IgG2b κ Isotype Control, FITC-CD4, PE-CD8, PE-IL17, APC-CD25, PE-Foxp3 (BD Biosciences, USA); RBPJ Inhibitor-1 (TargetMol, USA); Annexin V-FITC Apoptosis Detection Kit, Enhanced mitochondrial membrane potential assay kit (Beyotime, Shanghai, China).

### Bioinformatics prediction

The target genes regulated by miR-30b-5p were predicted by TargetScanHuman (http://www.targetscan.org/vert_71/). The Comparative Toxicogenomics Database (CTD, http://ctdbase.org/) was used to obtain uveitis-related disease targets with "uveitis" as the search term. The miR-30b-5p-target data set and the uveitis target data set were intersected to obtain the common genes between miR-30b-5p and uveitis using the Venny2.1 (https://bioinfogp.cnb.csic.es/tools/venny/index.html) online software tool. To mine the data of the direct or indirect regulatory relationship, we constructed a network between the potential targets of the miR-30b-5p and uveitis to analyze the overlapping targets’ protein–protein interactions (PPIs) by using the String 11.0 database (https://string-db.org/) and common targets were calculated in the R environment. The common targets between miR-30b-5p and uveitis were input into the Metascape database (https://metascape.org/gp/index.html) for gene ontology (GO) biological process and Kyoto Encyclopedia of Genes and Genomes (KEGG) pathway enrichment analyses, and the possible biological processes and signaling pathways involved in the target were obtained.

### In vivo experiments

#### Animals and grouping

The principles for the care and use of experimental animals in research strictly abided by the Guidelines for the Care and Use of Laboratory Animals published by the National Institutes of Health of China and the ARVO Statement on the Application of Animals in Ophthalmological and Visual Research. The experiments were approved by the Experimental Animal Management Committee of the Affiliated Hospital of Shandong University of Traditional Chinese Medicine (Number: AWE-2022-017). In this study, healthy female Lewis rats (6-week-old, 150-170g, Beijing Vital River Laboratory Animal Technology Co. Ltd, Beijing, China) were randomly divided into normal control (NC) group (n = 48), EAU group (n = 48), miR-30b-5p-carrying lentivirus injection group (miR-30b-5p group) (n = 48), and miR-30b-5p control vector-carrying lentivirus injection group (miR-30b-5p-N group) (n = 48). The rats from EAU, miR-30b-5p and miR-30b-5p-N groups were injected with chyle containing IRBP, TB, and CFA to induce EAU, while those in the NC group were injected with the same volume of TB and CFA chyle (without IRBP). On day 0 after immunization, miR-30b-5p-carrying lentivirus and miR-30b-5p control vector-carrying lentivirus (miR-30b-5p-N) were injected into the spleen of EAU rats in miR-30b-5p group and miR-30b-5p-N group, respectively (the amount of lentivirus injected was 5 × 10^7^ TU for each animal) for intervention. Prior to the experiments, the rats were adapted to the feeding environment for 1 week and were in the cycle of alternating day and night (12 h/12 h) under constant temperature (25 ± 2 ℃) and constant humidity (50 ± 10%).

#### Double luciferase experiment

The plasmid vector containing miR-30b-5p was combined with the relevant sites of the plasmid expressing the Notch1 and Dll4 luciferase reporter gene to create a mutant plasmid vector, and this mutant vector was then used to infect 293 T cells. The changes in the relative fluorescence value of the reporter gene were measured to verify whether Notch1 and Dll4 are target genes regulated by miR-30b-5p.

#### Clinical evaluation and histopathology

The ocular inflammation was observed with Genesis-D eye camera on day 12 after immunization and evaluated according to the grading criteria for rat ocular inflammation [12, 13]. The right eyeballs of the rats in each group on day 12 after immunization were fixed in eyeball fixation solution for 24h. Paraffin-embedded sections were prepared and dried for H&E staining. The pathological changes of the retina and ciliary body of rats in each group were observed, and the pathological scoring was assessed by the criteria proposed in the literature [14], and the score ranges from 0 (no inflammation) to 4 (maximum inflammation). In addition, the appearance and weight of the spleen and lymph nodes were photographed on day 12 after immunization.

#### RNA Sequencing

Eye tissue RNA Sequencing (RNA-Seq) was used to explore the transcriptional changes during inflammation in the EAU model after the miR-30b-5p intervention. Eye tissue RNA was extracted from the rats after immunization for 12 days in NC, EAU, and miR-30b-5p groups. Sequencing was performed on Illumina NovaSeq6000 by Xiuyue Biotechnology Co. Ltd. (Jinan, China).

#### Transmission electron microscopy

The spleen, lymph nodes and eye tissues were fixed in 4% glutaraldehyde at 4 ℃ for 2h on day 12 after immunization, followed by sectioning. The ultrastructural changes of different tissues were observed by transmission electron microscopy (TEM).

#### Quantitative PCR

RNA was extracted and reverse transcribed into cDNA by the reverse transcription kit (Sparkjade, Jinan, China). The primer sequences of each gene were listed in Supplement Table 1. The Q-PCR reaction conditions were as follows: 95 ℃, 5min for one cycle; 95 ℃, 20s, 57 ℃, 25s, 60 ℃, 25s for 45 cycles. After the detection was completed, the expression levels of Notch1, Dll4, IL-10, and IL-17 genes were calculated according to the 2^−ΔΔCt^ method.

#### ELISA

The protein concentration of each tissue was detected by the BCA protein assay kit in a 96-well plate (Wuxi NEST Biological Co. Ltd., Wuxi, China). The expression levels of Notch1, Dll4, IL-10, and IL-17 proteins were measured by the commercial ELISA kits (Jianglai Biotech., Shanghai, China).

#### Flow cytometry analysis

The approximately 1 × 10^6^ T lymphocytes were collected from each tissue, and stained with relevant fluorescence antibodies, followed by staining with FITC-CD4 and APC-CD25 for surface staining and PE-IL-17 and PE-Foxp3 for intracellular staining. Finally, the levels of Th17 Treg cells in the spleen, lymph nodes, and eye tissues were detected by a flow cytometer (BD FACSVerse™, USA). Meanwhile, an isotype control assay was performed to eliminate unwanted background cell staining.

#### Wes™ automatic protein expression analysis system

The protein concentration was quantified by the BCA protein assay kit in a 96-well plate (Wuxi NEST Biological Co. Ltd., Wuxi, China). Simple Western analyses were performed using the Wes™ automatic protein expression analysis system (Capillary Electrophoresis Immunoassay, ProteinSimple Santa Clara, CA). The primary antibodies (Notch1, ab52301, dilution 1:1000; Dll4, bs-5909R, dilution 1:2000) were prepared with antibody diluent II supplied by Proteinsimple Instruments. Further, 3 μL of the prepared sample was denatured at 95 ℃ for 5 min. After denaturation, 10 μL diluted primary and secondary antibodies were added to the Wes™ automatic protein expression analysis system. At the end of the operation, the fluorescence internal reference samples were checked and the data were analyzed.

#### Immunohistochemistry (IHC)

The spleen, lymph nodes, and eye tissues of the same position of the rats after immunization for 12 days in each group were sliced, and the thickness of the slice was vertically at 6 μm. The slices were placed at room temperature for 10–15 min and fixed with glacial acetic acid for 10–20 min. After each slice was washed with PBS, a drop of 3% H_2_O_2_ was dropped and placed at room temperature for 10 min, incubated with primary antibody (Notch1 1:400, Dll4 1:500) at 4 °C overnight, and then transferred the slices to room temperature for 30 min. Each slice was incubated with HRP-secondary antibody (1:200) and left at room temperature for 20 min after washing with PBS, received hematoxylin staining, rinsed with water for 1 min, dripped with 1% hydrochloric acid alcohol, saturated lithium carbonate solution for 1min, dehydrated with gradient alcohol for 2min. Finally, the slice was sealed with neutral gum and observed under the microscope (Nikon 55i, Japan).

#### Immunofluorescence staining

After initial perfusion with 4% paraformaldehyde in 0.1 mol/L of PBS (pH = 7.4), the eye tissues were enucleated and immediately embedded in CryoGlue (SLEE Medical GmbH, Germany), and then transferred to—80 °C. The eye tissues were sectioned vertically at 6 μm and were then blocked with 5% bovine serum albumin (BSA) containing 0.3% Triton X-100 in 0.01 M PBS at room temperature for 1 h. Next, these blocked sections were incubated with primary antibodies (Notch1, ab52301, dilution 1:1000; Dll4, bs-5909R, dilution 1:2000) diluted in PBS overnight at 4 °C. Then immunofluorescence was performed with secondary antibody (Goat anti-rabbit IgG H & L 555, ab150078, 1:400) for 2 h. The nucleus staining was performed with 4′,6-diamidino-2-phenylindole (DAPI, 1 μg/mL) counterstaining for 20 min. Finally, the sections were recorded under the LSM 780 laser confocal microscope (Zeiss, Germany) with a 63 oil-immersion objective.

#### PCR Array and ingenuity pathway analysis

The RNeasy Microarray Tissue Mini Kit (No. 73304) and the RNase-Free DNase Set (No. 79254) on-column DNase digestion process were used to remove a small amount of contamination in the RNA sample for RNA purification. According to the reverse transcription reaction system, the purified RNA was prepared into cDNA with RT^2^ First Strand kit, and the cDNA was mixed with RT^2^ SYBR Green MasterMix. The rat Notch signaling pathway RT^2^ Profiler PCR Array and Th17 reaction RT^2^ Profiler PCR Array G 384 (4 × 96) were used, and the ingenuity pathway analysis (IPA) system (version 42012434, Ingenuity Systems; Qiagen China Co., Ltd.) was used for bioinformatics analysis.

### In vitro experiment

#### Determination of multiplicity of infection (MOI) and T lymphocyte infection

The lentivirus stock solution was diluted with Enhanced Infection Solution (ENi.S) to three concentration titers of 1.0 × 10^8^ TU/mL, 1.0 × 10^7^ TU/mL, and 1.0 × 10^6^ TU/mL, respectively, and then 200 μL of conventional medium P(M) containing 50 μg/mL polybrene and ENi.S solution P(E) containing 50 μg/mL polybrene were prepared according to different Multiplicity of infection (MOI) levels (100, 50, 20, 10, 5, and 0, respectively), and then T lymphocytes from each tissue were seeded in cell culture dishes (NEST Biotechnology, Wuxi, China) and polarized under Th17 cell-polarizing cocktail consisting of rIL-1β (20 ng/mL), rIL-6 (60 ng/mL), rIL-23 (30 ng/mL), and TGF-β (2 ng/mL), anti-rat CD3 (5 μg/mL) and anti-rat CD28 (2 μg/mL). After transfection for 12 h, the mediums were replaced with fresh medium for further culture. After transfection for 72 h, the infection rate of the virus was analyzed by fluorescence microscopy, and the optimal MOI of infection was adopted for the T lymphocyte infection according to the results of the preliminary experiment. In this study, the lentivirus carrying miR-30b-5p and the negative control lentivirus of miR-30b-5p were added to the cultured cells of each group. After 72 h of cell culture, the lentivirus transfection effect was evaluated by a fluorescence microscope, and the cells in each group were collected for the subsequent experiments.

#### Q-PCR and ELISA

The spleen, lymph nodes (both sides of abdominal wall, armpit, groin), and left eye tissues from EAU rats were separated under aseptic conditions on day 12 after immunization. The T lymphocytes were isolated and purified by the lymphocyte isolation solution kit. After cell counting, the cells were transferred to a 6-well cell culture plate (Wuxi NEST Biological Co. Ltd., Wuxi, China), and then treated with miR-30b-5p-carrying lentivirus and miR-30b-5p control vector-carrying lentivirus, respectively. RNA and protein were extracted from the cells in each group after 72h, and then the expression of Notch1, Dll4, IL-10, and IL-17 within T lymphocytes was detected by Q-PCR and ELISA, respectively.

#### Flow cytometry analysis

T lymphocytes in each group were collected after miR-30b-5p-carrying lentivirus infection for 72 h. The levels of Th17, Treg cells, and apoptotic cells were detected by flow cytometry in each group.

#### In vitro cell isolation of patients with uveitis and functional validation

To verify the effect of inactivating the Notch pathway on Th cell differentiation and apoptosis in T cells from patients with uveitis, we used 2 μM RBPJ Inhibitor-1 (RIN1) to treat peripheral blood mononuclear cells (PBMCs) isolated from patients with uveitis to detect the changes in Th17/Treg, CD4^+^/CD8^+^ cell ratios, as well as changes in cell apoptosis and mitochondrial membrane potential levels.

### Statistical analysis

All the experiments were repeated three times. The data were expressed as the *mean* ± S.D. (standard deviation) and were analyzed by SPSS statistical software (SPSS for Windows, version 22.0, IBM-SPSS, Chicago, IL, USA). The Levene test was used to assay the variance, and the LSD test was used to compare the two groups. P < 0.05 was considered statistically significant. The IPA system (version 42012434) was used to perform the bioinformatics analysis. The right-tailed Fisher's exact test and unpaired t-test were used for the analysis and validation of DEGs, and P < 0.05 was considered to indicate a statistically significant difference.

## Results

### Bioinformatics analysis

**Fig. 1:**
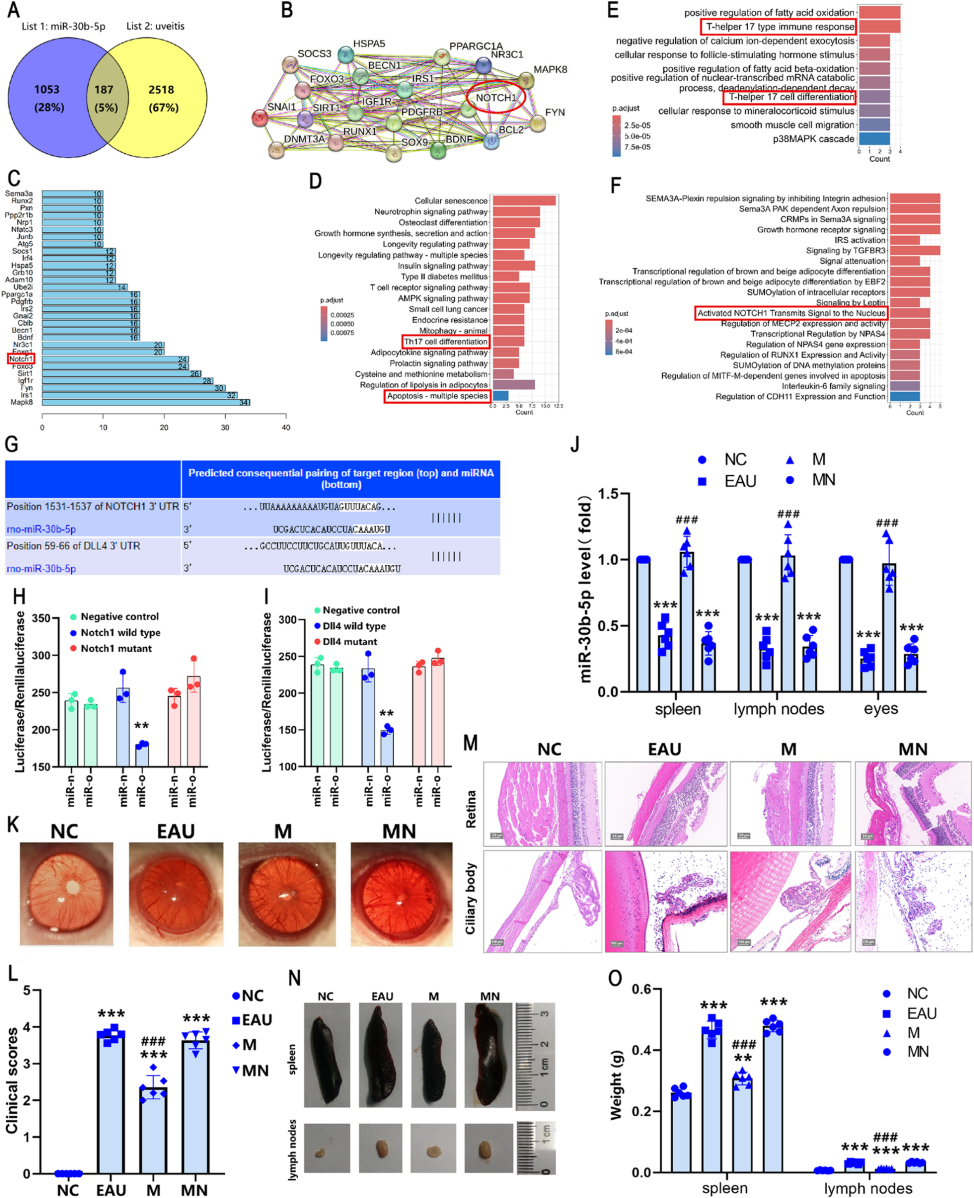
Bioinformatics analysis of the relationship between miR-30b-5p and EAU, and the validation of bioinformatics prediction and ocular inflammations, histopathological alterations and morphological changes, and the levels of miR-30b-5p in the spleen, lymph nodes, and ocular tissues in NC, EAU, miR-30b-5p, and miR-30b-5p-N groups on day 12 after immunization. **A** A total of 187 crossover genes between the miR-30b-5p and uveitis-related targets. **B** PPI network of crossover genes. **C** The summary of the top 30 core targets for the number of connected nodes. **D** The KEGG enrichment analysis. **E** The GO enrichment analysis. **F** The Reactome enrichment analysis. **G** The sequence of 3’-UTR where Notch1 and Dll4 mRNAs bound to miR-30b-5p. **H–I** Expression levels of Notch1 and Dll4 in 293T cells transfected with miRNA and plasmid vector. Compared with the negative control group, ^*^P < 0.05, ^**^P < 0.01. **J** The levels of miR-30b-5p in the spleen, lymph nodes, and ocular tissues in NC, EAU, miR-30b-5p, and miR-30b-5p-N groups on day 12 after immunization (n = 6). **K** Ocular inflammations of the rats after different treatments. **L** Clinical score of ocular inflammations (n = 6). **M** Histopathological alterations in the retina (bar = 50 μm) and ciliary body (bar = 100 μm). **N** Morphology of the spleen and lymph nodes. **O** Histogram of the change of the weight in spleen and lymph nodes (n = 6). Clinical scores and weight were presented as mean ± S.D. ^*^P < 0.05 compared with NC, ^**^P < 0.01 compared with NC, and ^##^*P* < 0.01 compared with EAU

Using the TargetScanHuman database, 1240 target genes were predicted for the miR-30b-5p (Supplement Table 2). Based on the CTD database, the disease name was set to uveitis and the influence score was set to ≥ 8, and 2705 final uveitis-related target genes were obtained (Supplement Table 3). A total of 187 crossover genes between the miR-30b-5p and uveitis-related targets were further obtained (Fig. 1A). The PPI network was shown in Fig. 1B. The summary of the top 30 core targets for the number of connected nodes was showed in Fig. 1C. The PPIs showed that miR-30b-5p can affect Notch 1 receptor, the representative component of Notch signaling, indicating that miR-30b-5p plays a crucial role in the biological processes in the pre-treatment of uveitis. The KEGG enrichment analysis (Fig. 1D) showed that most of the common targets between miR-30b-5p and uveitis were concentrated on physio pathological and immune-inflammatory courses, such as the Th17 cell differentiation, T cell receptor signaling pathway, apoptosis, and these pathways represent key approaches in the treatment of uveitis with miR-30b-5p. The GO enrichment analysis (Fig. 1E) showed that the enriched term mainly focused on biological processes, including T-helper 17 type immune response, T-helper 17 cell differentiation, and negative regulation of B cell apoptotic process, the molecular mechanism may be explained by the antiviral and anti-inflammatory effects of miR-30b-5p and this mechanism is closely related to the Th17 cell differentiation and apoptosis, indicating that miR-30b-5p may affect the Notch signaling pathway to further regulate Th17 cell differentiation. The Reactome enrichment analysis (Fig. 1F) showed that the common targets of miR-30b-5p and uveitis are concentrated in transcriptional regulation of cell differentiation, which involved activated Notch1 transmits signal to the nucleus, suggesting that the the action target of miR-30b-5p on uveitis was related to Notch1.

### miR-30b-5p negatively regulates the Notch1 and Dll4 expression

To confirm the targeted inhibitory effect of miR-30b-5p on the notch, we validated it through bioinformatics prediction and double luciferase assay. Bioinformatics prediction shows that Notch1 and Dll4 genes on the Notch signaling pathway are target genes regulated by miR-30b-5p. Using double luciferase expression report analysis, we found that the expression of the RLU value of wild-type Notch1 and Dll4 genes regulated by miR-30b-5p was significantly down-regulated compared with the negative control group. In contrast, the expression of the RLU value in the Notch1 and Dll4 gene mutant vector had no significant change (Fig. 1G–I), suggesting that both Notch1 and Dll4 are target genes regulated by miR-30b-5p.

## Results of in vivo experiments

### Clinical evaluation and histopathology

To explore the regulatory role of miR-30b-5p on the notch signaling pathway and its therapeutic effect on uveitis, we conducted functional validation through in vivo animal experiments. The levels of miR-30b-5p in the spleen, lymph nodes, and eye tissues of EAU rats injected with miR-30b-5p-carrying lentivirus were significantly increased on day 12 after immunization, while there was no significant difference in the levels of miR-30b-5p in spleen, lymph nodes and eye tissues of EAU rats injected with miR-30b-5p control vector-carrying lentivirus (Fig. 1J). The observation with a Genesis-D eye camera showed that compared with the rats of the NC group, the eyes of EAU rats and the miR-30b-5p-N group showed congestion and swelling on day 6 after immunization. On day 12 after immunization, the manifestation of the eyes showed severe inflammation, including severe congestion and expansion of iris blood vessels, closure of pupil membrane, and pus. In contrast, the ocular inflammation in the miR-30b-5p group was significantly reduced compared to the EAU and miR-30b-5p-N groups. We also noted that there were no ocular iris adhesion and pupillary membrane closure except for mild or moderate iris vascular congestion (Fig. 1K). The scores of the rats in EAU, miR-30b-5p, and miR-30b-5p-N groups were 3.82 ± 0.16, 2.53 ± 0.37, and 3.56 ± 0.31, respectively (Fig. 1L). Meanwhile, pathological examination showed that the ocular tissue structure in the NC group was intact. In EAU and miR-30b-5p-N groups, the eye tissue structure was disordered, the whole layer of the retina was destroyed, and a large number of inflammatory cells were observed in the ciliary body and retina. By contrast, the retina and the ciliary body of rats in the miR-30b-5p group showed only mild to moderate inflammatory cell infiltration, and there was no full-thickness destruction of retina and disorder of tissue structure (Fig. 1M). The size and weight of the spleen and lymph nodes in the EAU group were significantly greater than those in the NC group on day 12 after immunization, and the size and weight of spleen and lymph nodes reduced significantly after miR-30b-5p intervention, suggesting that miR-30b-5p can significantly promote the recovery of inflammation (Fig. 1N–O).

### RNA-Seq and data analysis

**Fig. 2:**
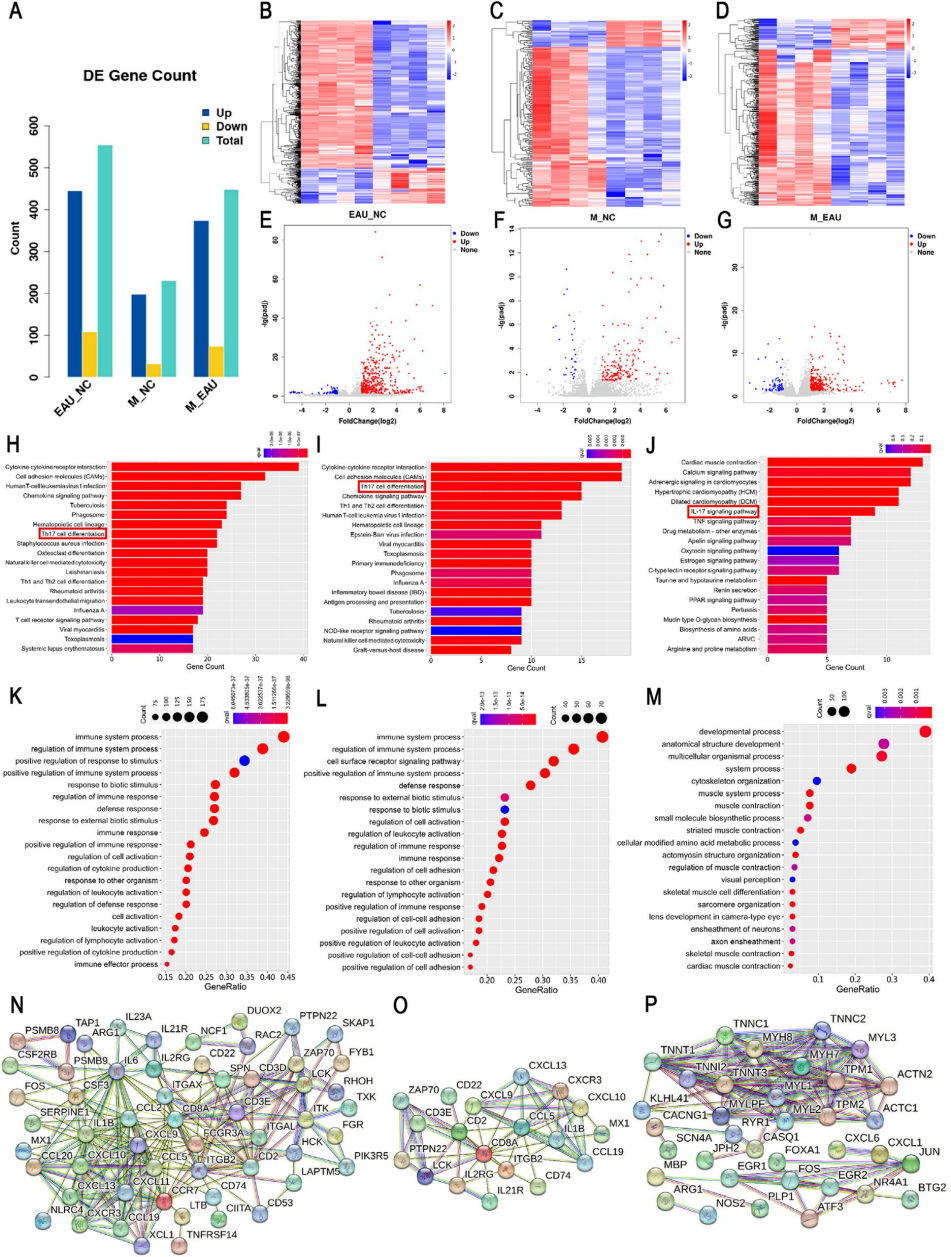
RNA-Seq and bioinformatics analysis. Screening differentially expressed genes by sequencing results of the whole transcriptome (**A**). Heatmap and volcano maps of differentially expressed genes (**B–G**). Visualization analysis of KEGG pathways, GO terms, and PPI network (**H–P**). NC vs. EAU (**B, E, H, K, N**), NC vs. M (**C, F, I, L, O**), M vs. EAU (**D, G, J, M, P**). M = miR-30b-5p group

The sequencing results of the whole transcriptome revealed that 554 DEMs were screened between NC and EAU groups, including 445 up-regulated genes and 109 down-regulated genes. 231 DEMs were screened between the NC and M groups, including 198 up-regulated genes and 33 down-regulated genes. 448 DEMs were screened between EAU and M groups, including 374 up-regulated genes and 74 down-regulated genes (Fig. 2A). A heatmap and volcano maps of DEMs showed that these mRNAs were significantly differentially expressed among NC group-, EAU group-, and M group-derived samples (Fig. 2B–G). A total of 20 KEGG pathways significantly enriched were identified among the groups, which were mainly involved in Th17 cell differentiation and IL-17 signal pathway (Fig. 2H–J). The functional annotation of the GO analysis revealed that these DEGs were mainly related to biological processes such as immune system process, regulation of immune system process, cell activation, immune response, and so on (Fig. 2K–M). In addition, we identified a large number of inflammatory chemokines (CCL2, CCL5, CCL19, CCL20, CXCL9, CXCL10, CXCL11, and CXCL13), inflammation-related genes (IL-6, IL-23A, IL-21R, and IL-2RA) and many immune-related pathway genes (CD2, CD5, CD22, CD40, and CD74) (Fig. 2N–P). Taken together, functional analysis revealed that the biological processes involved in the significantly expressed genes may be related to the Th17 cell differentiation and immunoregulation process.

### Transmission electron microscopy

**Fig. 3:**
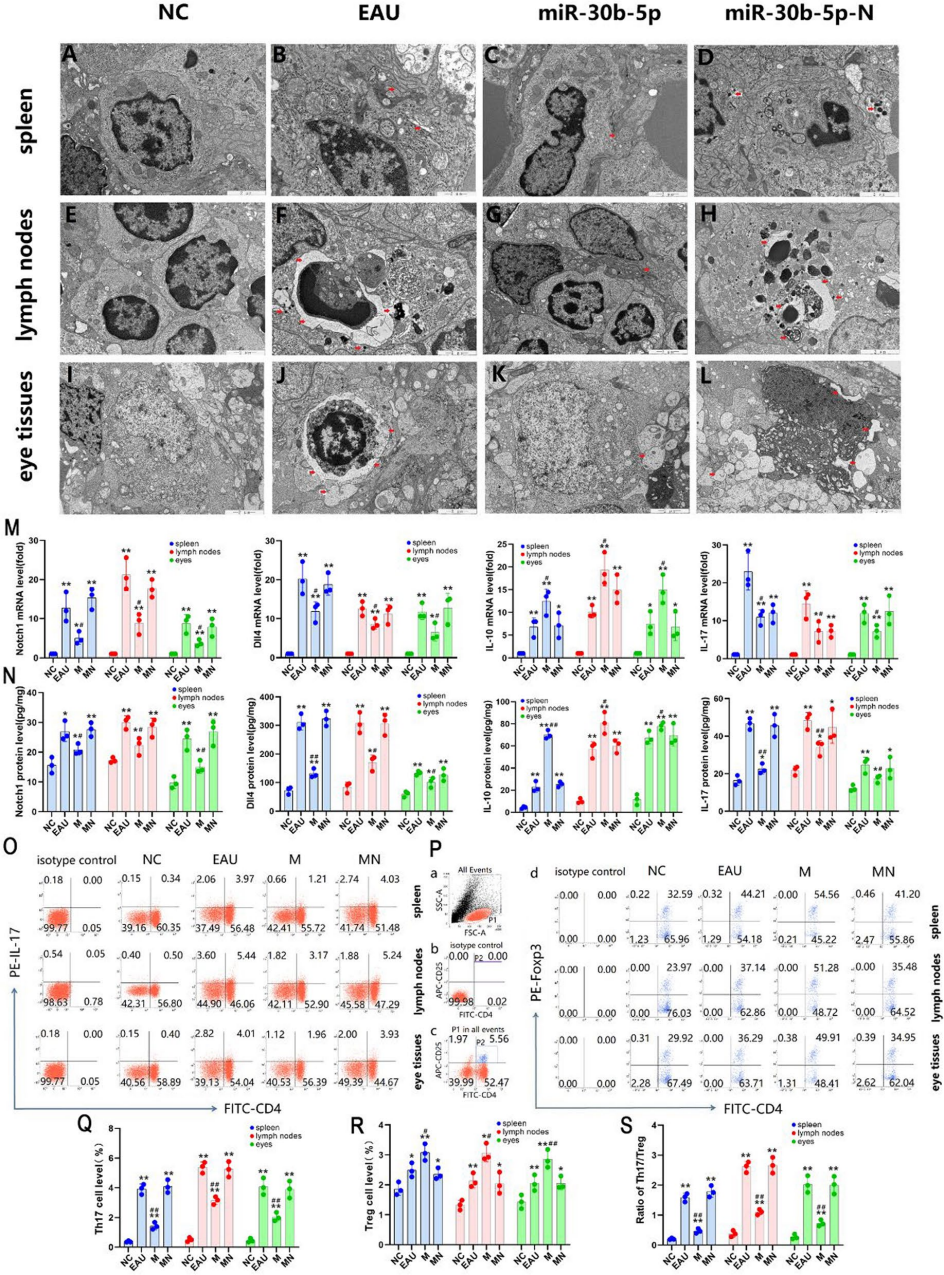
Ultrastructural images and changes of Notch1, Dll4, IL-10, IL-17, Th17, and Treg levels in spleen, lymph nodes, and eye tissues in NC, EAU, miR-30b-5p, and miR-30b-5p-N groups on day 12 after immunization. On day 12 after immunization, the ultrastructural changes of spleen, lymph node and eye tissues were observed by transmission electron microscopy. **A, E, I** NC groups; **B, F, J** EAU groups; **C, G, K** miR-30b-5p groups; and **D, H, L** miR-30b-5p-N groups. **M** Expression levels of Notch1, Dll4, IL-10, IL-17 mRNAs. **N** Expression levels of Notch1, Dll4, IL-10, IL-17 proteins. **O** Th17 cell frequencies. **P** Treg cell frequencies. a CD4^+^ T lymphocytes, b representative scatter-grams of the intracellular expression of Foxp3 within CD4^+^CD25^+^ T cells. CD4^+^CD25^+ ^T lymphocytes were gated in P1 of (b), (c) was from P1 of (a), and CD4^+^CD25^+^Foxp3^+^ T cells (Treg cells) were shown in the right-upper quadrant of (d). Changes of Th17 levels (**Q**), Treg levels (**R**), and Th17/Treg ratios (**S**) were showed in histograms. ^*^P < 0.05, ^**^ *P* < 0.01 compared with NC, and ^#^*P* < 0.05, ^##^*P* < 0.01 compared with EAU

The TEM analysis showed that the ultrastructure of the spleen, lymph nodes, and eyes in the NC group showed uniform cytoplasm, clear boundary membrane, large nucleus, rich heterochromatin, and uniform distribution. There were abundant mitochondria in the cytoplasm, and the cells possessed clear nuclear membranes and clear cristae structures. Nevertheless, the ultrastructure of the tissue in EAU and miR-30b-5p-N groups showed vacuole and membrane damage of mitochondria, widening of perinuclear space, expansion of rough endoplasmic reticulum, swelling and necrosis of more cells, pyknosis of mitochondria and dense matrix. The plasma membrane of some cells disappeared and the nucleus pyknosis. Mitochondrial maturation was excessive. In the miR-30b-5p group, the ultrastructure of each tissue was clear, the cytoplasmic core membrane was clear, and the mitochondria did not appear vacuole, membrane damage, or obvious nuclear pyknosis (Fig. 3A–L).

### Determination of Notch1, Dll4, IL-10 and IL-17 mRNA levels

Q-PCR analysis showed that the expression of Notch1, Dll4, IL-10, and IL-17 mRNAs in the spleen, lymph nodes, and eyes from EAU and miR-30b-5p-N groups at 12 days after immunization were significantly more than those from NC group (all *P* < 0.05). Meanwhile, the mRNA levels of Notch1, Dll4, and IL-17 in the miR-30b-5p group were significantly less than those in the EAU group, whereas the mRNA levels of IL-10 were significantly elevated (all *P* < 0.05). However, there was no significant difference in Notch1, Dll4, IL-10, and IL-17 gene levels between miR-30b-5p-N and EAU groups (*P* > 0.05) (Fig. 3M).

### Measurement of Notch1, Dll4, IL-10 and IL-17 protein levels by ELISA

The Notch1, Dll4, IL-10, and IL-17 protein expression levels in spleen, lymph nodes, and eye tissues in EAU and miR-30b-5p-N groups were significantly more than those in the NC group (all *P* < 0.05), and the Notch1, Dll4 and IL-17 protein levels in spleen, lymph nodes and eyes in miR-30b-5p group were also significantly elevated. However, the levels of Notch1, Dll4, and IL-17 proteins in the spleen, lymph nodes and eyes in the miR-30b-5p group were significantly less than those in the EAU group, and the level of IL-10 was significantly increased (all *P* < 0.05). Moreover, we also noted that there was no significant difference between EAU and miR-30b-5p-N groups (*P* > 0.05) (Fig. 3M). These results showed that miR-30b-5p could effectively reduce the expression of Notch signaling pathway-related molecules and effectively inhibit the differentiation of Th cells.

### Flow cytometry

After the determination with flow cytometry, we noted that Th17 levels (Fig. 3N, O) in the spleen, lymph nodes, and eyes in EAU and miR-30b-5p-N groups were significantly higher than those in the NC group, and the ratio of Th17/Treg increased significantly, showing the unbalanced state (all *P* < 0.05). Meanwhile, the ratios of Th17/Treg in the spleen, lymph nodes, and eyes in the miR-30b-5p group significantly lower than that in the EAU group, and the ratio tended to restore balance. However, the ratios of Th17/Treg cells in the spleen, lymph nodes, and eyes in the miR-30b-5p-N group were similar to those of the EAU group (*P* > 0.05) (Fig. 3P, Q, R).

### Determination of Notch1 and Dll4 protein levels by automatic protein expression analysis system

**Fig. 4:**
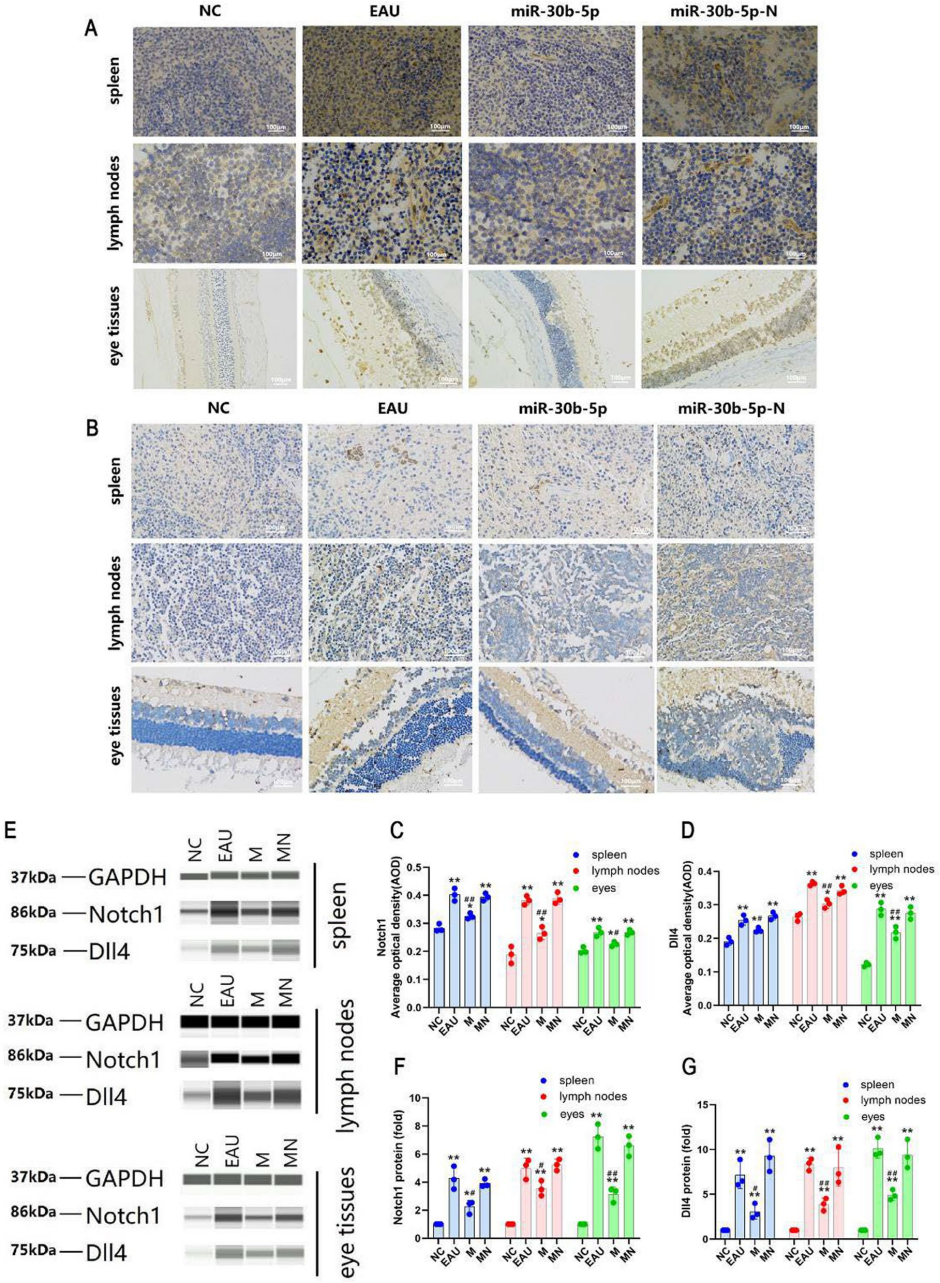
Expression of Notch1 and Dll4 proteins in spleen, lymph nodes, and eye tissues of NC, EAU, miR-30b-5p, and miR-30b-5p-N groups detected by Wes™ automatic protein expression analysis system and IHC assay. **A, B** The levels of Notch1 and Dll4 proteins. **C–D** Histogram of changes in Notch1 (**C**) and Dll4 (**D**) proteins. **E** The levels of Notch1 and Dll4 proteins. **F–G** Histogram of optical density analysis in Notch1 (**F**) and Dll4 (**G**) proteins. ^*^P < 0.05, ^**^P < 0.01; compared with EAU group, ^#^ *P* < 0.05, ^##^ *P* < 0.01. For IHC assay, magnification = 400 × , bar = 100 μm

Wes™ automatic protein expression analysis system showed that the expression levels of Notch1 and Dll4 proteins in the spleen, lymph nodes, and eyes of the miR-30b-5p group were significantly down-regulated compared with those in the EAU group on day 12 after immunization (*P* < 0.05); nevertheless, there was no significant difference in the expression of Notch1 and Dll4 proteins in the spleen, lymph nodes, and eye tissues between miR-30b-5p-N and EAU groups (Fig. 4A, B), indicating that miR-30b-5p could efficiently reduce the expression of Notch signaling pathway-related molecules.

### Expression of Notch1 and Dll4 in rat spleen, lymph nodes, and eyes by IHC

The results showed that the expression levels of Notch1 (Fig. 4C) and Dll4 (Fig. 4D) proteins in the spleen, lymph nodes, and eye tissues in the miR-30b-5p group were reduced than those in the EAU group on day 12 after immunization (*P* < 0.05) (Fig. 4E). However, we also noted that there was no significant difference in the expression of Notch1 and Dll4 proteins in the spleen, lymph nodes, and eye tissues in the miR-30b-5p-N group compared with those in the EAU group.

### Enhanced expression of Notch1 and Dll4 proteins by immunofluorescence staining

**Fig. 5:**
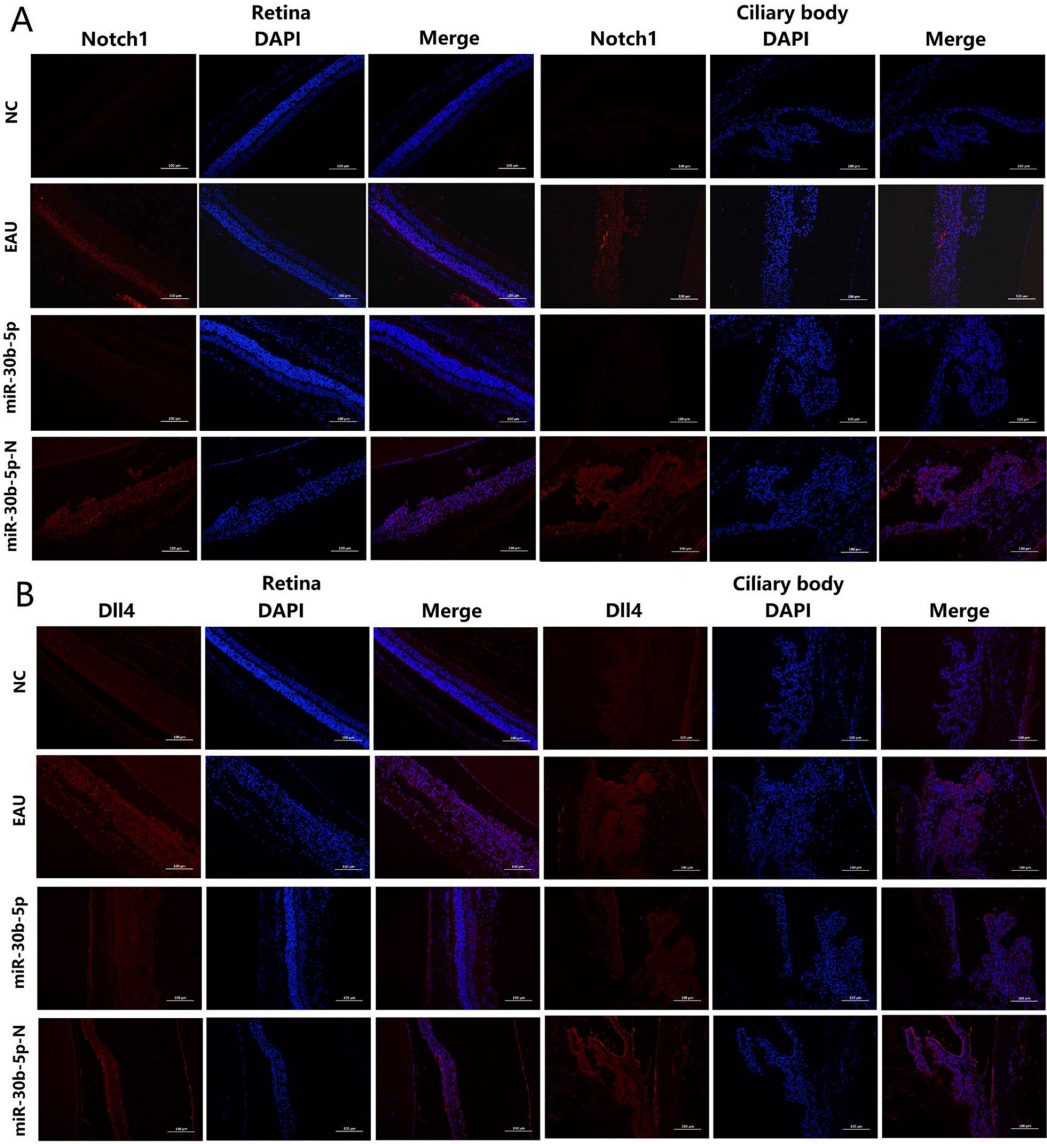
The expression of Notch1 and Dll4 proteins in retina and ciliary body in NC, EAU, miR-30b-5p, and miR-30b-5p-N groups detected by immunofluorescence staining. Cell nuclei stained with DAPI showed blue fluorescence, and Notch1 (**A**) and Dll4 (**B**) showed red fluorescence (magnification = 200 × , bar = 100 μm)

In this study, immunofluorescence staining of Notch1 (Fig. 5A) and Dll4 (Fig. 5B) proteins showed red fluorescence. Compared with the NC group, Notch1 and Dll4 proteins were highly expressed in the retina and ciliary body of the EAU group, however, the red fluorescence intensity in the retina and the ciliary body was significantly weakened after the miR-30b-5p intervention, especially in the ciliary body in the miR-30b-5p group, the staining of Notch1 protein could not or only detected in traces, suggesting the decreased expression levels of Notch1 and Dll4 proteins. However, there was no decrease in red fluorescence intensity in the retina and ciliary body of the miR-30b-5p-N group, indicating miR-30b-5p could efficiently inhibit the activation of the Notch signaling pathway in EAU.

### DEGs of Notch signaling and Th17 response by PCR array analysis

The expression levels of 84 genes related to the Notch signal pathway regulation network were analyzed by the Notch signal pathway PCR array, including Notch pathway ligands, receptors, and related genes, Notch target genes, apoptosis, cell cycle, transcription-regulation related genes, and genes involved in cell regulation, proliferation, and differentiation. There were 20 differentially expressed mRNAs in the EAU group compared with the NC group, including 4 up-regulated genes and 16 down-regulated genes, and there were 13 differentially expressed mRNAs in the miR-30b-5p group compared with the EAU group, including 11 up-regulated genes and 2 down-regulated genes (Supplement Fig. 1A). Similarly, the results of the Th17 cell response PCR array assay showed that compared with NC group, there were 38 DEGs in the EAU group, including 18 up-regulated genes and 20 down-regulated genes; and compared with EAU group, there were 26 DEGs in the miR-30b-5p group, including 4 up-regulated genes and 22 down-regulated genes (Supplement Fig. 1B).

### Enriched canonical pathways by IPA

**Fig. 6:**
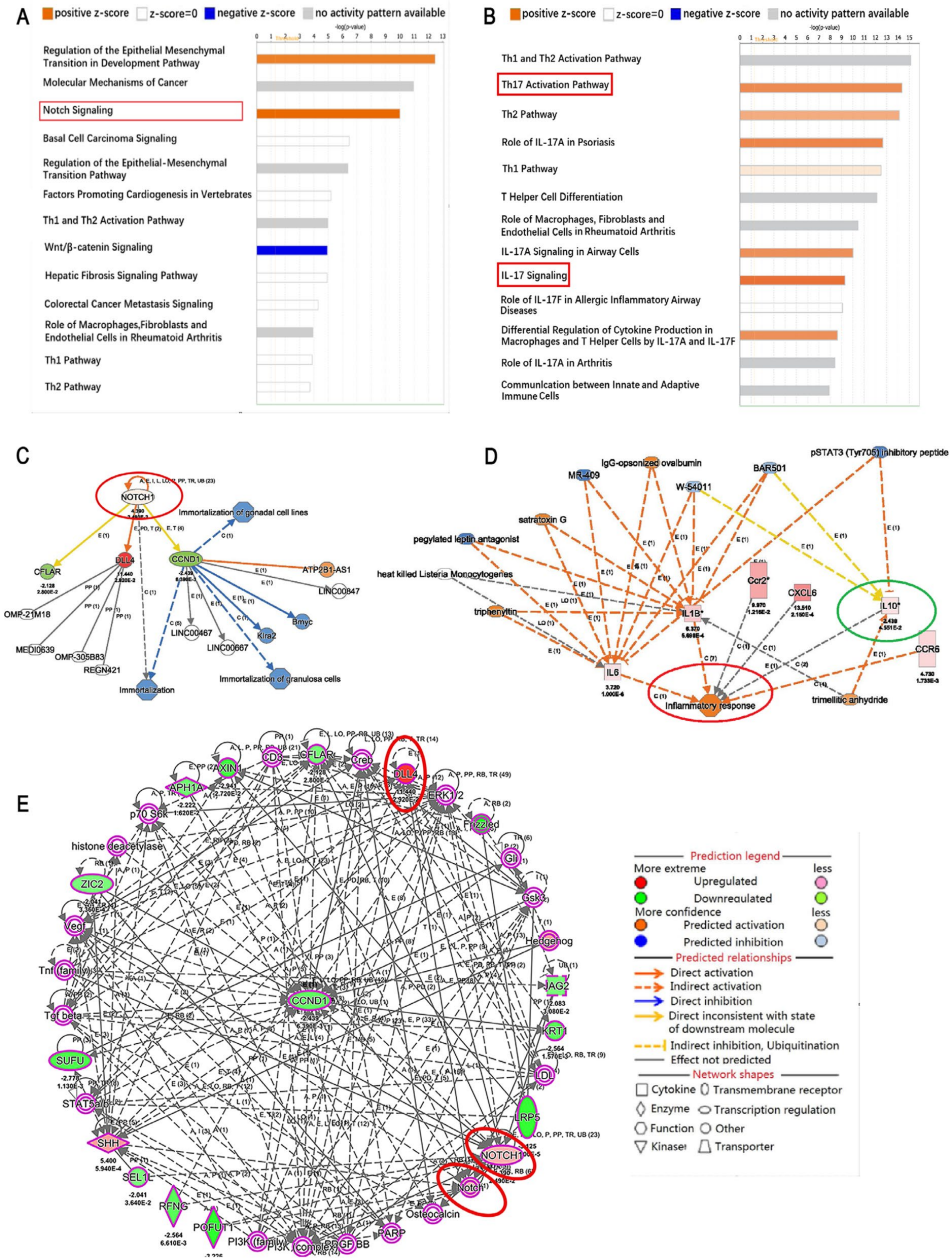
Summary of the bioinformatics annotation analyzed by IPA. **A, B** Enriched canonical pathways detected by IPA. The top 13 enriched canonical pathways were identified using the minus log (P-value) > 2 threshold in the EAU group. The enrichment results showed that Notch signaling and the Th17 activation pathways were apparently activated. **C, D** Regulatory effect analysis. The regulatory effect analysis showed the possible pathways of upstream regulatory networks and downstream functions. The prediction of upstream regulators and downstream functions was performed using IPA. Solid lines represent a direct interaction between the two genes, whereas dotted lines represent an indirect relationship. The length of a line reflects the strength of reported evidence supporting the node-to-node relationship. The shapes of the nodes represent the different known biological roles of these molecules, as shown in the lower right inset. **E** Molecular network analysis. The molecular network interaction analysis reflects the interaction between molecules in the dataset

The top categories as ranked following the minus log(P-value) were shown in Fig. 6. A total of 43 enriched canonical pathways were identified using the minus log (P-value) > 2 thresholds in the EAU group. The top 13 of the 43 representative pathways were found to be associated tightly with the Notch signaling pathway and were shown in Fig. 6A, the enrichment results showed that the Notch signaling pathway was activated. Regarding Th17 response, we found that a total of 90 enriched canonical pathways were identified using the threshold of minus log > 2. We found top 13 of the 90 representative pathways were tightly associated with the Th17 reaction (Fig. 6B), and the Th17 activation pathway was also obviously activated.

### Regulatory effects of DEGs by IPA

The regulatory effect analysis showed the possible pathways of upstream regulatory networks and downstream functions involved in the DEGs [15]. The consistency score is an indicator used to describe the causal consistency of the upstream regulatory factor in the network, the dataset, and dense connection metric between diseases and functions [16]. The Prediction of upstream regulators and downstream functions performed using IPA were shown in Fig. 6C, D. The results showed that Notch1 and Dll4 were upstream regulatory network molecules, of which Notch1 was the upstream regulatory molecule of Dll4, and both of them were up-regulated. In addition, IL-10 as an anti-inflammatory factor and IL-6 as a pleiotropic cytokine were upstream regulatory network molecules, and both of them were up-regulated. The inflammatory response was the downstream effect. IL-6 and IL-10 can directly or indirectly regulate the inflammatory response.

### Molecular network analysis

The interaction molecular network analysis reflects the interaction between molecules in the dataset. In this study, the results of the interaction molecular network analysis showed that Notch1 and Dll4 were involved in the interaction network involving 35 related molecules, and both of them were up-regulated (Fig. 6E).

## Results of in vitro experiments

### Q-PCR and ELISA

**Fig. 7:**
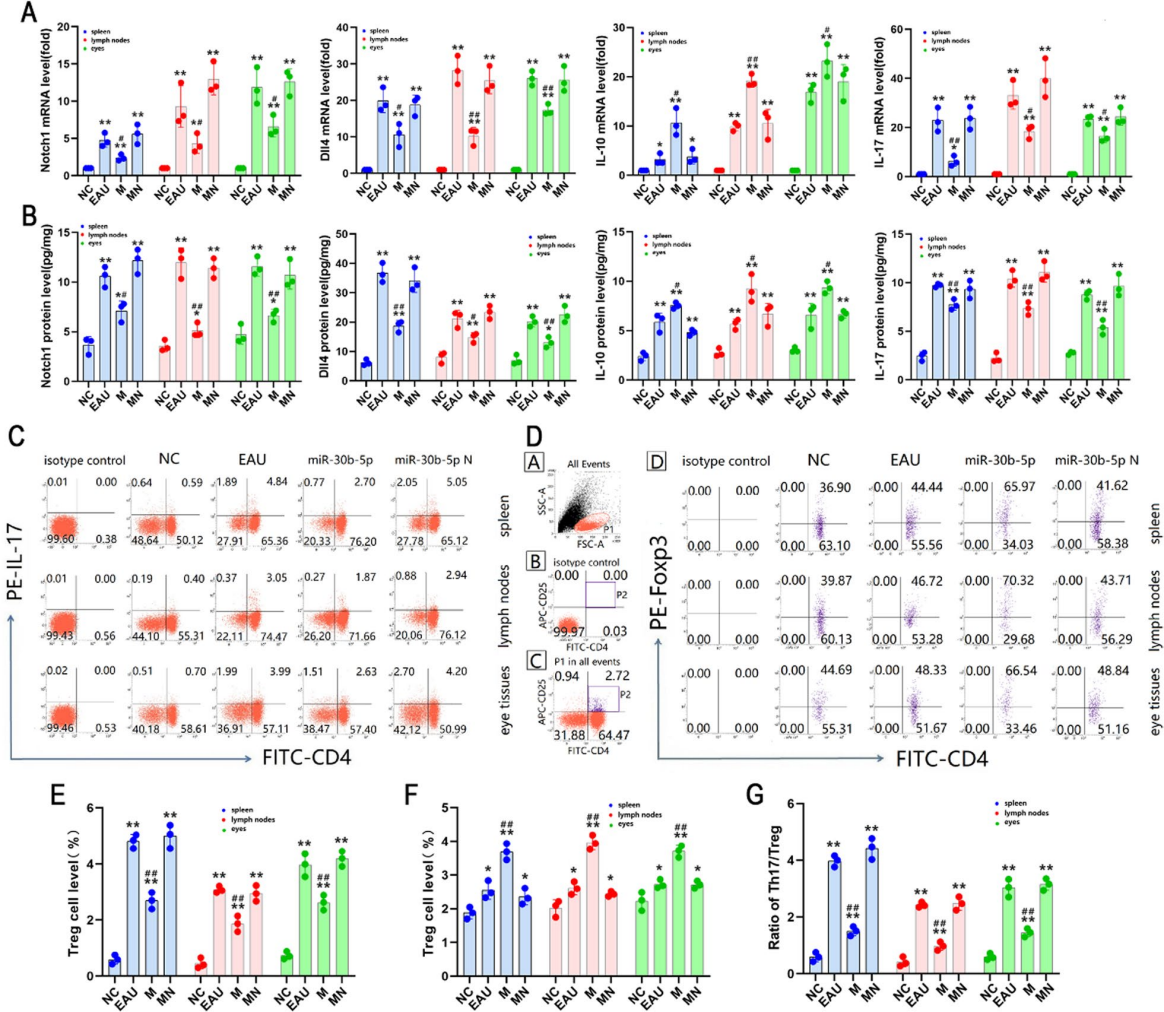
Changes of Notch1, Dll4, IL-10, IL-17, Th17, and Treg levels in T lymphocyte after miR-30b-5p-carrying lentivirus transfection in vitro for 72 h. T lymphocytes isolated from the spleen, lymph nodes, and eye tissues in NC, EAU, miR-30b-5p, and miR-30b-5p-N groups were determined by flow cytometry to assess the levels of Th17 and Treg cell lineages. **A, B** Expression levels of Notch1, Dll4, IL-10, IL-17 mRNAs and proteins. **C** Th17 cell frequencies. **D** Treg cell frequencies. a CD4^+^ T lymphocytes, b representative scatter-grams of the intracellular expression of Foxp3 within CD4^+^ CD25^+^ T cells. CD4^+^ CD25^+ ^T lymphocytes were gated in P1 of (b), (c) was from P1 of (a), and CD4^+^CD25^+^Foxp3^+^ T cells (Treg cells) were shown in the right-upper quadrant of (d). Changes in Th17 levels (**E**), Trig levels (**F**), and Th17/Treg ratios (**G**) were showed in bar graphs. ^*^P < 0.05, ^**^ *P* < 0.01 compared with NC, and ^#^*P* < 0.05, ^##^*P* < 0.01 compared with EAU

The results of Q-PCR showed that the expression of Notch1, Dll4, IL-10, and IL-17 genes in T lymphocytes of the spleen, lymph nodes, and eyes in EAU and miR-30b-5p-N groups was significantly more than those in NC group after lentivirus transfection in vitro for 72 h (all *P* < 0.05). Meanwhile, the mRNA levels of Notch1, Dll4, and IL-17 in T lymphocytes in the miR-30b-5p group were significantly lower than those in the EAU group, whereas the mRNA levels of IL-10 were significantly higher than those in the EAU group (all *P* < 0.01). However, there was no significant difference in Notch1, Dll4, IL-10, and IL-17 gene levels between the miR-30b-5p-N and EAU groups (*P* > 0.05) (Fig. 7A). Similarly, the protein levels of Notch1, Dll4, IL-10, and IL-17 in cells and the supernatants in each group were also consistent with the expression trend of gene level after miR-30b-5p-carrying lentivirus transfection, and the protein levels of Notch1, Dll4, IL-10 and IL-17 in EAU and miR-30b-5p-N groups were significantly higher than those in the NC group (all *P* < 0.05). Moreover, we also noted that there was no significant difference between EAU and miR-30b-5p-N groups (*P* > 0.05). In contrast, the expression of Notch1, Dll4, and IL-17 protein in cells and the supernatants decreased significantly, whereas the level of IL-10 increased significantly (all *P* < 0.05) (Fig. 7A).

### Differentiation of Th cells and apoptosis

**Fig. 8:**
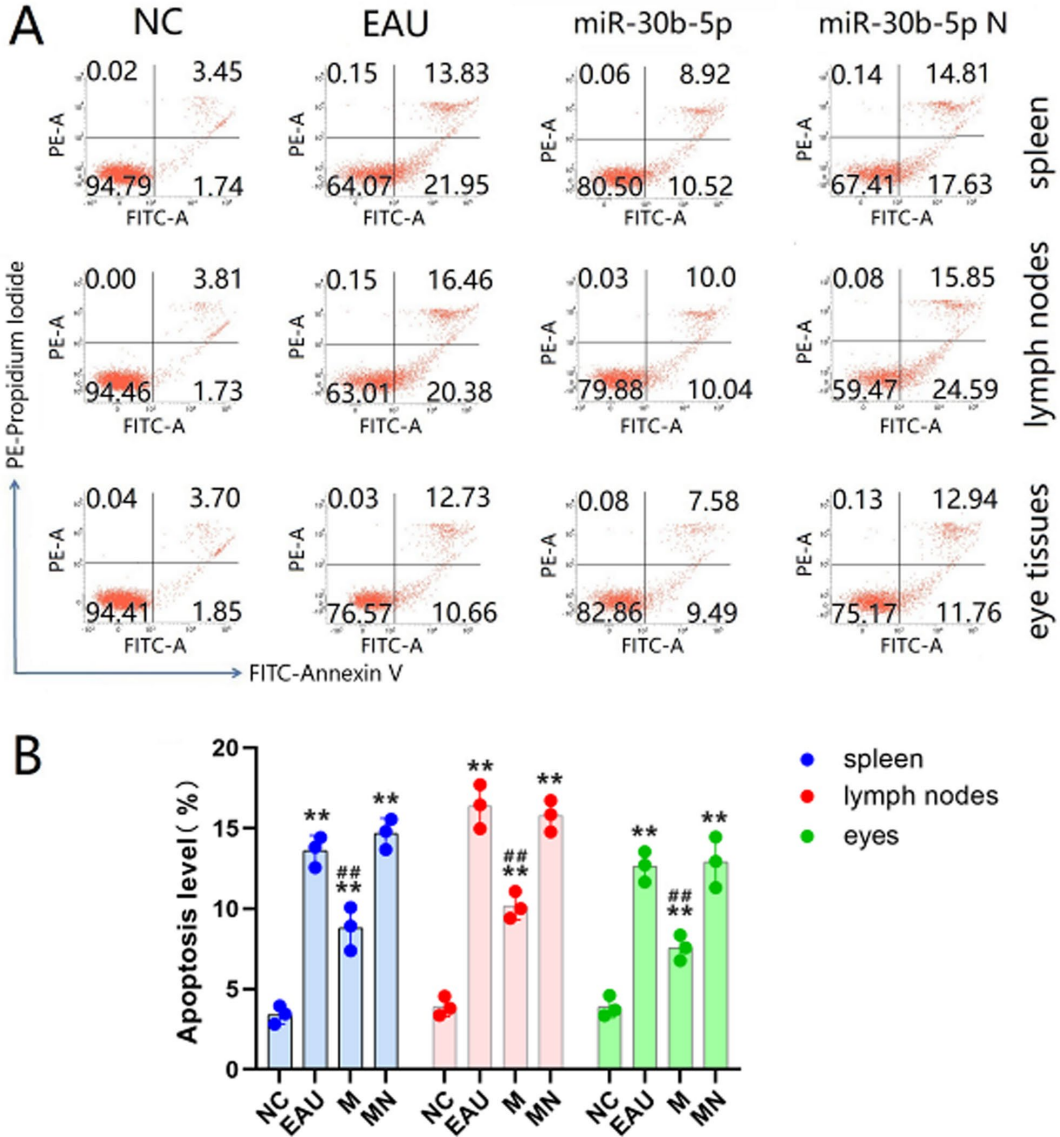
The apoptotic levels in T lymphocytes after lentivirus transfection in vitro for 72 h. To assess the apoptotic levels, T lymphocytes were isolated from the spleen, lymph nodes, and eye tissues in NC, EAU, miR-30b-5p, and miR-30b-5p-N groups, and the apoptotic levels of T lymphocytes were determined by flow cytometry. **A** The third quadrant (upper right) of each axis represents the level of apoptosis in each group. (**B**) Histogram analysis of apoptotic levels. ^*^P < 0.05, ^**^ *P* < 0.01 compared with NC, and ^#^*P* < 0.05, ^##^*P* < 0.01 compared with EAU

Compared with the NC group, the Th17 frequencies in EAU and miR-30b-5p-N groups elevated significantly, accompanied by the elevated proportion of Th17/Treg (all *P* < 0.05). Meanwhile, the proportion of Th17/Treg in the miR-30b-5p group was significantly lower than that in the EAU group, and it tended to be balanced. Nevertheless, there was no significant difference between miR-30b-5p-N and EAU groups (Fig. 7B–F). And the level of apoptosis in the miR-30b-5p group was significantly lower than the level of the EAU group (all *P* < 0.05) (Fig. 8). These results showed that miR-30b-5p could effectively reduce apoptosis and the differentiation of naive T cells into Th17 cells, thereby reducing the proportion of Th17/Treg.

### In vitro cell isolation of patients with uveitis and functional validation

**Fig. 9:**
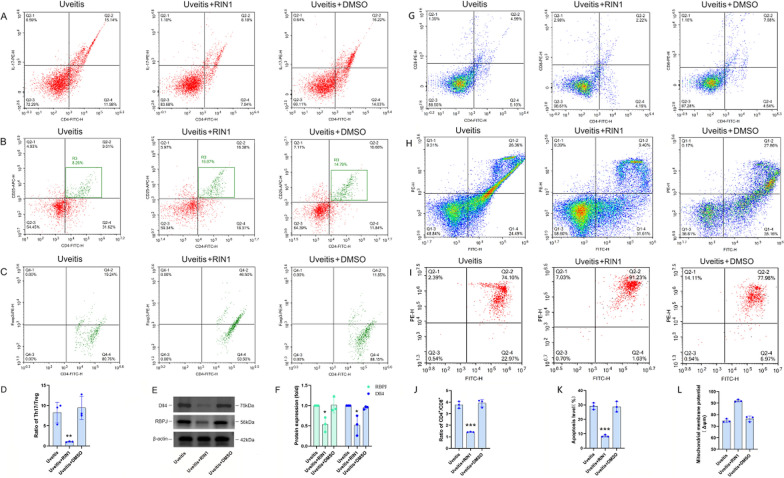
Changes of the ratio of Th17/Treg, Dll4 and RBPJ proteins, the ratio of CD4^+^/CD8^+^, the level of apoptosis, and mitochondrial membrane potential in T lymphocyte from Peripheral blood of patients with uveitis after RIN1 and DMSO in vitro for 24 h. **A** Th17 cell frequencies. **B, C** Treg cell frequencies. CD4^+^ CD25^+ ^T lymphocytes were gated in P1 of (**B**), (**C**) was from R3 of (**B**), and CD4^+^CD25^+^Foxp3^+^ T cells (Treg cells) were shown in the right-upper quadrant of (**C**). **D** Histogram analysis of ratio of Th17/Treg. **E** The levels of Dll4 and RBPJ proteins. **F** Histogram of optical density analysis in Dll4 and RBPJ proteins. **G** CD4^+^, CD8^+^cell frequencies. **H** Histogram analysis of ratio of CD4^+^/CD8^+^. **I** The apoptotic levels. **J** Histogram analysis of apoptotic levels. **K** The mitochondrial membrane potential levels. **L** Histogram analysis of mitochondrial membrane potential levels. ^*^P < 0.05, ^**^ *P* < 0.01, ^***^ *P* < 0.001 compared with uveitis group

In addition, we more directly demonstrated the effect of Notch pathway inactivation on the Th cells differentiation and apoptosis in uveitis by isolating in vitro cells from patients with uveitis and intervening with RBPJ inhibitors. In vitro cell studies have found that the expression of Dll4 and RBPJ in T lymphocytes of patients with uveitis is significantly reduced after treatment with 2μM RIN1 for 24h (Fig. 9E–F), and the proportion of Th17/Treg (Fig. 9A–D), CD4^+^/CD8^+^ (Fig. 9G, J), and the apoptotic cell level (Fig. 9H, K) were decreased, whereas the mitochondrial membrane potential level (Fig. 9I, L) was increased, and there was no significant difference after treatment with equal amount of DMSO, indicating that inactivating the Notch pathway can significantly attenuate Th17 cell differentiation, CD4^+^/CD8^+^ cell ratio and cell apoptosis, and elevate mitochondrial membrane potential levels in lymphocytes of patients with uveitis.

## Discussion

The Notch signaling pathway is an evolutionarily conserved signaling pathway in the development of multicellular organisms. It plays an important role in regulating autoimmune diseases and inflammatory responses. Its spatiotemporal expression specifies a variety of cellular events including proliferation, apoptosis, differentiation, and stem cell self-renewal [17–19]. Chronic hepatitis C patients who received Notch signaling pathway inhibitors can block Notch signaling, reduce the expression of Th17 cells and RORγt, and down-regulate the secretion of cytokines by Th17 cells, indicating that Notch signaling can maintain the function of Th17 cells in chronic HCV infection and further inhibit the regulatory function of Tregs [20]. Th17/Treg balance plays a critical role in maintaining the homeostasis of the peripheral immune microenvironment. Yu et al. [21] found that the activation of Notch signaling plays an important role in Th17/Treg imbalance in immune thrombocytopenia (ITP). In addition, recent studies have revealed that Notch signaling can also play an essential role in bone development and stabilization of the intraosseous environment [22–25]. Notch signaling is also related to peripheral T-cell activation and effector cell differentiation. Th17 and Treg cells as well as Th1 and Th2 cells may contribute to the development of ITP, thus, inhibition of Notch signaling may be a potential immunomodulatory strategy for patients with ITP. In addition, Weng et al. [26] showed that anti-Dll4 antibodies could inhibit the differentiation of Th17 cells in asthmatic mice. Therefore, the Notch signaling pathway plays a fundamental role in maintaining Th17/Treg balance and in regulating Th17 cell differentiation.

As a kind of refractory eye disease that seriously endangers human health, uveitis is usually caused by autoimmune disorders. It mostly occurs in young adults, mainly manifested as chronic or recurrent ocular inflammation. Yu et al. [27] successfully solved the specific immunotherapy of EAU mice by inducing autoantigen-specific Treg cells in vivo without damaging the whole host T cell immunity. Their study has potential significance for the treatment of patients with autoimmune uveitis. Yang et al. revealed that the overexpression of macrophage migration inhibitory factor (MIF) can activate the Notch signaling pathway, and then aggravate ocular inflammation in EAU mice. MIF antagonist ISO-1 can reduce intraocular inflammation and inhibit the differentiation of Th1 and Th17 in EAU mice, suggesting that the MIF-notch axis plays a role in the pathogenesis of EAU [28].

miRNAs are a kind of functional small RNA molecules that can negatively regulate the expression of target genes at the post-transcriptional level. It can regulate the expression of target genes by RNA interference (RNAi). The abnormal expression of miRNAs plays an important role in the pathogenesis of uveitis. Behçet disease (BD) is a refractory inflammatory disease with unknown causes. Its clinical manifestation is nongranulomatous recurrent panuveitis. Recently, miRNAs have been determined as significant regulators in autoimmunity. Abnormal expression and dysfunction of miRNAs can damage the function of the immune system and promote autoimmune diseases [29]. Clinical studies have shown that the enhanced activation of Notch signaling may be one of the pathogenesis of BD. In active BD patients, Notch pathway activation and Th17 response are enhanced, and miR-23b is significantly reduced. In patients with active Behcet's disease, blocking the Notch signaling pathway can preferentially inhibit the differentiation of Th17, and the decreased expression of miR-23b may be involved in the activation of Notch signaling in BD [30]. Jadideslam G et al. [31] found that the expression of miR-21 and miR-146b decreased significantly, while the expression of miR-326 increased significantly in BD patients. The level of miR-21 in patients with severe ocular involvement and miR-326 in patients with uveitis and severe ocular involvement were elevated. Thus, the level of miR-326 can be used as a biomarker to predict uveitis and severe ocular lesions in patients with BD. Meanwhile, our previous studies have also revealed that the decreased level of miR-30b-5p is closely related to the pathogenesis of uveitis by regulating IL-10 and TLR4, and miR-223-3p serves as an important regulator in M1 macrophage polarization and pyroptosis, thereby alleviating the inflammatory response in uveitis [10, 32].

At present, the changes of cytokines secreted by Th cells, especially by Th17 and Treg cell lineages, are closely related to the pathogenesis of uveitis [33]. Lee et al. [34] demonstrated that differences in gene expression between T cell subsets are greater than those between healthy controls and BD patients. Tang et al. [35] found that after inhibiting the differentiation of CD4^+^ T cells, the expression of IFN-γ, IL-17, and TNF-α decreased, and the production of IL-10 increased, contributing to the restoration of EAU. Kaabachi et al. [36] found that CD4^+^ T cells and monocytes stimulated by IL-26 promoted the production of Th17-related cytokines and inhibited the production of Treg-related cytokine IL-10. The elevation of CD8^+^ T cell CD11c level in BD patients suggests that the upregulation of CD11c in CD8^+^ cells may be related to the pathogenesis of BD [37].

A variety of cells and signal molecules in the immune system can maintain the body tissue in a healthy physiological state. Autoimmune diseases are characterized by the destruction of the balance of the immune system, and most of them are accompanied by inflammatory reactions against self-antigens and related tissue damage [38–42]. The ratio of CD4^+^/CD8^+^ in vitreous humor has a higher diagnostic value for granulomatous uveitis [43, 44]. Bing et al. [45] revealed that AS101, a small non-toxic, immunomodulator, can regulate autoimmune T cells by inhibiting the polarization of retina-specific T cells to Th1 or Th17 lineage and promoting Treg production, meanwhile, Li et al. [46] also found that elevated levels of monocyte chemotactic protein (MCP)-1, IL-17 and IFN-γ and decreased level of IL-10 occurred in aqueous humor in EAU rats, indicating the disturbed immune microenvironment is related to the differentiation of CD4^+^ T cells into Th17 and Treg cells. As a significant effector cytokine, IL-10 can drive disruption of immune mediators in autoimmune diseases [47], and the disturbed immune microenvironment could also lead to unbalanced expression levels of IL-17 and IL-10. In addition, clinical studies have also shown that the disruption of Th17/Treg balance plays an important role in the occurrence of uveitis. Th17 cells from BD patients can secrete IL-17 and IFN-γ at the same time under stimulation conditions, thereby activating the Th17 and Th1 responses of BD patients [48]. The clinical remission of uveitis patients is closely related to the increased TGF-β and IL-10 levels in serum and is positively related to Treg cells. The IFN-γ, IL-17A, and IL-22 levels in serum were significantly reduced compared with those of the patients with active uveitis [49]. Our previous studies found that inhibition of Notch signaling by N-(N-(3,5-Difluorophenacetyl-L-alanyl))-S-phenylglycine t-Butyl Ester (DAPT) can efficiently decrease Th17 cell response, downregulate the expression of Notch1, DLL4, IL-17 and the transcription of RORγt, reduce Th17 levels and restore the Th17/Treg balance [50], and Longdan Xiegan Decoction (LXD) also can efficiently inhibit Th17 cell differentiation, decrease the inflammatory cytokine expression, and restore the Th17/Treg balance by inhibiting the activation of the Notch signaling pathway in rats with EAU [51]. Therefore, the proportion of Th17/Treg cells can regulate the immune response and the pathogenesis of uveitis.

In this study, we found that the Th17 levels in the spleen, lymph nodes, and eye tissues in EAU and the miR-30b-5p-N groups were highly increased compared with those of the NC group, and the ratio of Th17/Treg cells also increased significantly. The miR-30b-5p-carrying lentivirus treatment can significantly decrease the ratio of Th17/Treg, leading to a balanced Th17/Treg ratio. However, the ratio of Th17/Treg cells in the spleen, lymph nodes, and eye tissues of rats in the miR-30b-5p-N group did not show obvious change, indicating that miR-30b-5p can effectively reduce the Th17 differentiation levels in EAU rats and correct the imbalance of Th17/Treg ratios, thereby contributing to the recovery of uveitis (Graphical abstract). In addition, we also found that inactivating the Notch pathway can ameliorate the imbalance of Th17/Treg differentiation and apoptosis of T lymphocytes in patients with uveitis, indicating that inactivating the Notch signaling pathway ameliorates the Th17/Treg and CD4^+^/CD8^+^ balance, contributing to the therapeutic role of miR-30b-5p on uveitis.

## Conclusion

The present study shows that the elevation of Notch1 and Dll4 levels and the activation of the Notch signaling pathway can regulate the differentiation of CD4^+^ T cells into Th17 cells and disturb the balance of Th17/Treg cells, participating in the inflammatory progression of uveitis. miR-30b-5p can negatively regulate the expression of Notch1 and Dll4 in EAU rats, significantly inhibit the activation of the Notch signaling pathway and the differentiation of Th cells into Th17 cells, promote the balance of the proportion of Th cells, and thus exert the therapeutic effect on experimental autoimmune uveitis from the perspective of "balanced and holistic view". Our findings provide basic research and theoretical basis support for the clinical application of microRNAs in the treatment of uveitis, paving the way for microRNAs treating inflammatory diseases in clinical practice.

## Clinical perspectives

- (i) Uveitis is an autoimmune eye disease that poses a significant risk of blindness, particularly in young adults. The disruption of the immune microenvironment plays a crucial role in the development of uveitis. Therefore, investigating both the local and systemic immune balance in this condition is a critical area for treatment research.
- (ii) miR-30b-5p has been shown to effectively inhibit the activation of Notch signaling and modify the Th17/Treg ratio. Clinical and laboratory studies indicate that inactivating the Notch pathway significantly reduces Th17 cell differentiation, alters the CD4^+^/CD8^+^ cell ratio, and decreases cell apoptosis. As a result, miR-30b-5p demonstrates a strong ability to suppress Notch signaling activation and Th17 cell differentiation, offering therapeutic potential for treating uveitis from a "balanced and holistic" perspective.
- (iii) The findings presented here provide essential research insights and theoretical support for the clinical application of microRNAs in managing uveitis.

## Supplementary Information

Supplementary Material 1: Analysis of the response genes related to upstream and downstream involved in Notch signal transduction pathway. Total RNA was extracted, and then detected by Notch signaling pathway RT² Profiler PCR Array and Th17 reaction RT² Profiler PCR. (a, b) Scatter diagram; (c, d) Volcano plot; (e, f) Heat map. The scatter plot compares the standardized expression of each gene on the PCR array between the two selected groups. The method is to draw each other to quickly show large gene expression changes. Volcano map identifies significant gene expression changes by drawing log2 of gene expression fold changes on the X axis and statistical significance on the Y axis. By aggregating a large number of data results, the heat map more clearly shows the frequency or density of spatial data.

Supplementary Material 2.

Supplementary Material 3.

Supplementary Material 4.

## Data Availability

Data in this study is available upon request from the corresponding author at dadonggene@163.com.

## Abbreviations

miRNAs: MicroRNAs
EAU: Experimental autoimmune uveitis
Th: T helper
RIN1: RBPJ inhibitor-1
Th2: T helper 2 cell
qPCR: Quantitative fluorescent PCR
DCs: Dendritic cells
cDNA: Complementary deoxyribonucleic acid
IRBP: Interphotoreceptor retinoid-binding protein
TB: Tuberculin
CFA: Complete Freund’s adjuvant
GO: Gene ontology
KEGG: Kyoto encyclopedia of genes and genomes
RNA-Seq: RNA Sequencing
TEM: Transmission electron microscopy
IHC: Immunohistochemistry
MOI: Multiplicity of infection
PBMCs: Peripheral blood mononuclear cells
MIF: Migration inhibitory factor
RNAi: RNA interference
BD: Behçet disease
MCP: Monocyte chemotactic protein
DAPT: N-(N-(3,5-Difluorophenacetyl-L-alanyl))-S-phenylglycine t-Butyl Ester
LXD: Longdan Xiegan Decoction
