# Supplementary material for: Therapeutic effect of miR-30b-5p-loaded lentivirus on experimental autoimmune uveitis via inhibiting Notch signaling activation
Source: J Transl Med. 2025 Apr 10;23:426. doi: 10.1186/s12967-025-06438-x (PMC11987260; doi:10.1186/s12967-025-06438-x)
Supplement: Supplementary file 1 — Supplementary Material 1: Analysis of the response genes related to upstream and downstream involved in Notch signal transduction pathway. Total RNA was extracted, and then detected by Notch signaling pathway RT² Profiler PCR Array and Th17 reaction RT² Profiler PCR. (a, b) Scatter diagram; (c, d) Volcano plot; (e, f) Heat map. The scatter plot compares the standardized expression of each gene on the PCR array between the two selected groups. The method is to draw each other to quickly show large gene expression changes. Volcano map identifies significant gene expression changes by drawing log2 of gene expression fold changes on the X axis and statistical significance on the Y axis. By aggregating a large number of data results, the heat map more clearly shows the frequency or density of spatial data. [file 12967_2025_6438_MOESM1_ESM.docx]

**Supplement Figure**

**
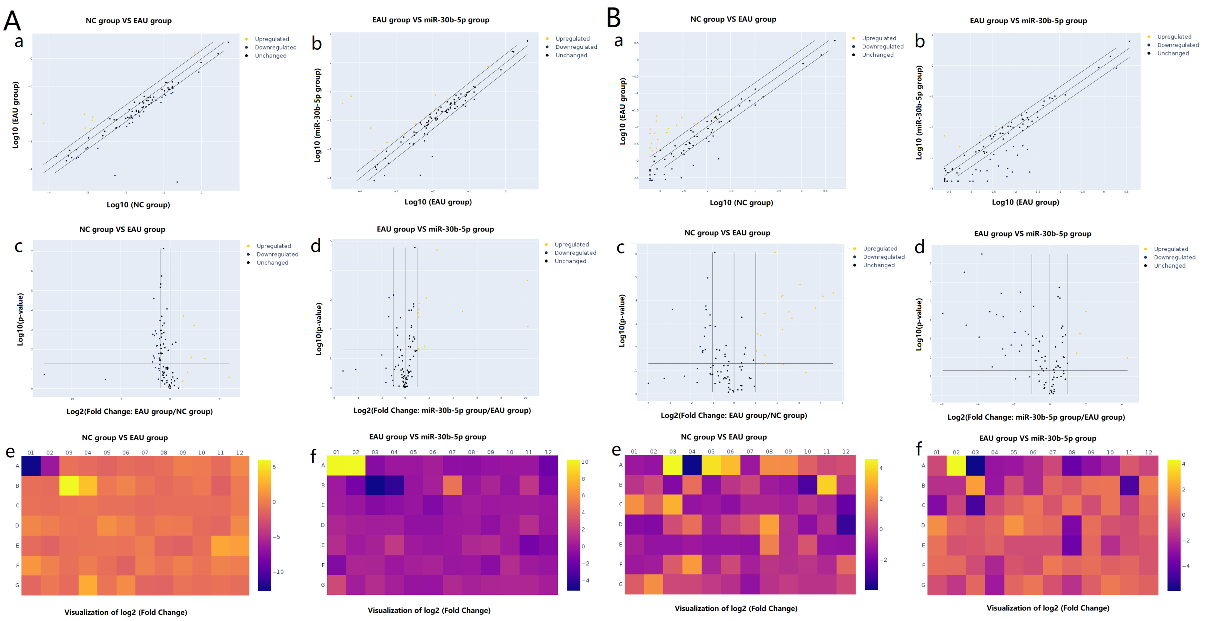
**

Supplement Figure 1 Analysis of the response genes related to upstream and downstream involved in Notch signal transduction pathway. Total RNA was extracted, and then detected by Notch signaling pathway RT² Profiler PCR Array and Th17 reaction RT² Profiler PCR. (a, b) Scatter diagram; (c, d) Volcano plot; (e, f) Heat map. The scatter plot compares the standardized expression of each gene on the PCR array between the two selected groups. The method is to draw each other to quickly show large gene expression changes. Volcano map identifies significant gene expression changes by drawing log2 of gene expression fold changes on the X axis and statistical significance on the Y axis. By aggregating a large number of data results, the heat map more clearly shows the frequency or density of spatial data.
