# Supplementary material for: Therapeutic effect of miR-30b-5p-loaded lentivirus on experimental autoimmune uveitis via inhibiting Notch signaling activation
Source: J Transl Med. 2025 Apr 10;23:426. doi: 10.1186/s12967-025-06438-x (PMC11987260; doi:10.1186/s12967-025-06438-x)
Supplement: Supplementary file 2 — Supplementary Material 2. [file 12967_2025_6438_MOESM2_ESM.docx]

**Table 3 Primer sequences for gene expression analysis**

| Gene Primer Sequences |
| --- |
| GAPDH Forward: 5'-GACCACAGTCCATGACATCACT-3',  Reverse: 5'-TCCACCACCCTGTTGCTGTAG-3'  Notch1 Forward: 5'-atggccccacctgcagacaagatg-3'  Reverse: 5'-ggcacggcaggcacagcgatag-3';  DLL4 Forward: 5'-CAAGAATAGCGGCAGTGGTCGTAA-3',  Reverse: 5'-GTAGCGCAGTCTTGTGAGGGTGTT-3';  IL-10 Forward: 5'-ttccatccggggtgacaataa-3',  Reverse: 5'-ttctgggccatggttctctgc-3';  IL-17 Forward: 5'-ttgctgctactgaacctggag-3',  Reverse: 5'-gcatggcggacaatagag-3' |
|  |
